# Electrophoresis in nanochannels: brief review and speculation

**DOI:** 10.1186/1477-3155-4-12

**Published:** 2006-11-20

**Authors:** Fabio Baldessari, Juan G Santiago

**Affiliations:** 1 Department of Mechanical Engineering, Stanford University, Stanford, CA, USA

## Abstract

The relevant physical phenomena that dominate electrophoretic transport of ions and macromolecules within long, thin nanochannels are reviewed, and a few papers relevant to the discussion are cited. Sample ion transport through nanochannels is largely a function of their interaction with electric double layer. For small ions, this coupling includes the net effect of the external applied field, the internal field of the double layer, and the non-uniform velocity of the liquid. Adsorption/desorption kinetics and the effects of surface roughness may also be important in nanochannel electrophoresis. For macromolecules, the resulting motion is more complex as there is further coupling via steric interactions and perhaps polarization effects. These complex interactions and coupled physics represent a valuable opportunity for novel electrophoretic and chromatographic separations.

## Background

Recent advances in nano-scale fabrication techniques allow for novel experimentation of the role of fluidic systems in analysis, detection, and separation of chemical and biological agents [1-9]. Electric fields can be used to drive flow, move analytes, and separate ionic species in nanometer sized channels. Insightful theoretical and numerical explorations of the physics of electrokinetically-driven flows inside channels with dimensions comparable to the electric double-layer date back more than 40 years [10-16]. Most of these have centered on the bulk/neutral liquid flow and the advective and electromigration components of current in such channels. In the last few years, experimental work has turned to a more systematic probing of the behavior of so-called surface conduction in nanochannels [2,17], and to species-dependent ion transport[3,4,18]. In this note we briefly review a few interesting recent reports in the field of electrophoresis in nanochannels, and offer some speculations as to research directions and potential opportunities for new functionality. We want to emphasize that we present a selected number of physical phenomena that are relevant to electromigration of analyte ions in long, thin nanochannels (e.g., for separation), and the discussion below is by no means all-inclusive of the research in the nanochannel and/or nanopore field. Furthermore, when we cite and describe specific results or studies we have chosen to reference only selected publications as examples rather than providing a complete listing of the relevant work that has appeared in print.

## Nanochannel physics

Electrophoresis in nanochannels is characterized by the dominant presence of the electrical double layer (EDL) that is formed spontaneously at the interface between a solid and an electrolyte. Surface charge is shielded by counter-ions from the electrolyte. Part of these counter-ions are believed to condense on the surface (reducing the effective surface charge density), while another portion remains solubilized and diffuse. The characteristic thickness of the diffuse layer is the Debye length as determined by a local balance of electromigration toward the surface and diffusion away from the surface. This Debye length is typically formulated as λD=(εkBT/e2∑j=1Nzj2njb)1/2
 MathType@MTEF@5@5@+=feaafiart1ev1aaatCvAUfKttLearuWrP9MDH5MBPbIqV92AaeXatLxBI9gBaebbnrfifHhDYfgasaacH8akY=wiFfYdH8Gipec8Eeeu0xXdbba9frFj0=OqFfea0dXdd9vqai=hGuQ8kuc9pgc9s8qqaq=dirpe0xb9q8qiLsFr0=vr0=vr0dc8meaabaqaciaacaGaaeqabaqabeGadaaakeaacqaH7oaBdaWgaaWcbaGaemiraqeabeaakiabg2da9maabmaabaWaaSGbaeaacqaH1oqzcqWGRbWAdaWgaaWcbaGaemOqaieabeaakiabdsfaubqaaiabdwgaLnaaCaaaleqabaGaeGOmaidaaOWaaabmaeaacqWG6bGEdaqhaaWcbaGaemOAaOgabaGaeGOmaidaaOGaemOBa42aa0baaSqaaiabdQgaQbqaaiabdkgaIbaaaeaacqWGQbGAcqGH9aqpcqaIXaqmaeaacqWGobGta0GaeyyeIuoaaaaakiaawIcacaGLPaaadaahaaWcbeqaaiabigdaXiabc+caViabikdaYaaaaaa@4BEF@, where ε is the medium permittivity, *k*_*B*_*T *the thermal energy, *e *is the electron charge, and ∑j=1Nzj2njb
 MathType@MTEF@5@5@+=feaafiart1ev1aaatCvAUfKttLearuWrP9MDH5MBPbIqV92AaeXatLxBI9gBaebbnrfifHhDYfgasaacH8akY=wiFfYdH8Gipec8Eeeu0xXdbba9frFj0=OqFfea0dXdd9vqai=hGuQ8kuc9pgc9s8qqaq=dirpe0xb9q8qiLsFr0=vr0=vr0dc8meaabaqaciaacaGaaeqabaqabeGadaaakeaadaaeWaqaaiabdQha6naaDaaaleaacqWGQbGAaeaacqaIYaGmaaaabaGaemOAaOMaeyypa0JaeGymaedabaGaemOta4eaniabggHiLdGccqWGUbGBdaqhaaWcbaGaemOAaOgabaGaemOyaigaaaaa@3B5A@ is a summation of the bulk electrolyte ion density (*z*_*j *_and njb
 MathType@MTEF@5@5@+=feaafiart1ev1aaatCvAUfKttLearuWrP9MDH5MBPbIqV92AaeXatLxBI9gBaebbnrfifHhDYfgasaacH8akY=wiFfYdH8Gipec8Eeeu0xXdbba9frFj0=OqFfea0dXdd9vqai=hGuQ8kuc9pgc9s8qqaq=dirpe0xb9q8qiLsFr0=vr0=vr0dc8meaabaqaciaacaGaaeqabaqabeGadaaakeaacqWGUbGBdaqhaaWcbaGaemOAaOgabaGaemOyaigaaaaa@30E8@ are respectively valence number and number density). Bulk motion of the liquid in a nanochannel, electroosmosis, is created by ion drag upon application of a tangential electric field which drives the motion of diffuse counterions. Electrophoresis is also effected: the observable drift velocity of all mobile ions in the system. Below we describe phenomena that influence electrophoresis of dilute solutes, including analytes. We concentrate mostly on analyte species concentration low enough such that their presence does not appreciably disturb the background electrolyte dynamics that determine the characteristics of the EDL and velocity fields.

Nanometer-scale channels require a new view of electrophoretic motion, even in cases where the continuum assumptions are thought to hold. This is because observable motion of ions in nanochannels is not just explainable by the interaction of the external/axial field and the solvent (and a linear superposition of a uniform solvent velocity), but is a result of the complex coupling between these ions and the EDL. The EDL introduces not only non-uniform motion of the bulk/neutral solvent but also large non-uniform transverse electric fields. In the case of macromolecules and order 10 nm channels, observable motion is also coupled to steric interactions with walls. Consider the typical transverse field in an EDL, 10^7 ^V/m, [19] which tends to migrate counter ions toward the wall (and co-ions away from the wall). The timescale for such migration is order 10^-7 ^s for small ions. Thus an equilibrium is establishes between transverse electromigration and diffusion, resulting in transverse concentration gradients in a nanochannel with length scales that are particular to each ion. This analyte-specific length scale of species *s*, λ_*S*_, is a function of its valence number (*z*_*S*_), the electrolyte Debye length, λ_*D*_, zeta potential, and temperature: for example, under the Debye-Huckle approximation for small wall potential and for sample ions with valence significantly larger than background ions, *z*_*S *_> *z*, λ_*S *_ε -λ_*D*_(*k*_*B*_*T*/*ze*ζ)/(*z*_*S*_/*z*), where ζ is the wall potential (assumed negative), and *z *is, for example, the ion valence in an otherwise symmetric background electrolyte. This makes the interaction of each low concentration analyte ion and the solvent a "personal issue" between it and the velocity and electric fields set up by the background of all other ions in the system. Direct evidence of this are the experiments of Pennathur and Santiago which showed that the transport of analyte ions is determined not just by (bulk) ion mobility but is a function of ion valence, EDL thickness, and surface charge density[3,4]. To the best of our knowledge this is the first time that this length scale (λ_*S*_) is explicitly defined. Its derivation and how it can be helpful to interpret experimental data will be discussed in one of our upcoming publications.

Another unique feature of nanochannels is the relevant regimes of Taylor-type dispersion (i.e., dispersion due to velocity gradients) of small dilute analytes that do not interact (adsorb or desorb) with the walls. Nanochannels are typically fabricated with high aspect ratio cross sections (*w*/*h *values range from 5 to 250 [3,9], where *h *and *w *are the channel depth and width respectively). The time scale for neutral-species diffusion across the channel depth, τ_*h*_~*h*^2^/*D *(*D *is diffusivity) is therefore typically short compared to that across channel width (τ_*w*_~*w*^2^/*D*). Concentration gradients across the channel depth quickly equilibrate under flow. Ajdari *et al*. [20] points out that dispersion in shallow-channels with smooth spanwise height distributions should be controlled by the product κ_*l*_·Pew2
 MathType@MTEF@5@5@+=feaafiart1ev1aaatCvAUfKttLearuWrP9MDH5MBPbIqV92AaeXatLxBI9gBaebbnrfifHhDYfgasaacH8akY=wiFfYdH8Gipec8Eeeu0xXdbba9frFj0=OqFfea0dXdd9vqai=hGuQ8kuc9pgc9s8qqaq=dirpe0xb9q8qiLsFr0=vr0=vr0dc8meaabaqaciaacaGaaeqabaqabeGadaaakeaacqWGqbaucqWGLbqzdaqhaaWcbaGaem4DaChabaGaeGOmaidaaaaa@31BE@, where *Pe*_*w *_= *wU*/*D *is the Peclet number based on the channel width instead of the channel height, and κ_*l *_is non-dimensional function that depends on the specific shape of the cross-section (defined in equation (7)) of Ajdari *et al*.). The consequence of this is that dispersion in channels with order 10 nm depth is not necessarily trivial if their width is of order 10 μm or larger. In wide, shallow channels dispersion due to spanwise velocity gradients occurs at rates which are not negligible compared to spanwise diffusion (which tends to homogenize the solute plug). Statistical sampling of the solute in the width (spanwise) direction is therefore less efficient and leads to increased dispersion over that of an idealized, infinitely-wide channel with the same depth scale. For charged analytes, the question of predicting dispersion coefficients and dispersion rates becomes more difficult. Here, cross-flow diffusion is constrained by the presence of the electric field in the EDL. We can therefore expect dispersion in nanochannels to be a function of the coupling between the non-uniform velocity and the fast equilibrium between two-dimensional cross-flow diffusion and cross-flow electromigration.

A more comprehensive model can become complicated very quickly. For example, consider that the two most important material properties in electrokinetics, viscosity and permittivity, are each believed to vary strongly along the EDL[19,21]. In microchannel electrophoresis, such complexities can be "buried" by just basing convection-diffusion-electromigration models of the bulk region (outside the EDL) on the empirically observed slip velocity created by the EDL. Nanochannels are a different matter as all ions now spend a significant fraction of time migrating through the EDL and its non-uniform material properties. Is the observable electrophoretic motion now not a function of such non-uniformities? Do net axial transport measurements contain useful information regarding these non-uniformities? Further, consider the case of even weakly adsorbing/desorbing ions. Typically, adsorption/desorption kinetics can be neglected if length is large enough such that diffusion acts on a time scale that is much longer than that for adsorption/desorption kinetics: this is the case when the Damkohler number is large, *Da *= *k*_*ads*_*h*/*D *>> 1 (here *k*_*ads *_is the rate constant for first order irreversible adsorption kinetics). From this simple scaling, we estimate that, for nanochannels of 40 nm depth, adsorption rates as low as 10^-4 ^s will noticeably influence net transport. This implies that many electrophoresis experiments may actually turn into chromatographic separations when performed in nanochannels. Can this be exploited for new functionality? Are chiral separations possible? Support for these statements comes from the work of, for example, Garcia *et al*. who observed separation of a neutral dye (rhodamine B) from a dye with valence -2 (Alexa 488) in *SiO*_2 _channels with relevant dimension that varied between 35 and 200 nm[1]. In their experiments, the electrokinetic radius (*h*/λ_*D*_) was varied between 2 and 12, and they attributed the separation to two mechanisms: (1) electrostatic repulsion of Alexa 488 from the negatively-charged walls, coupled with a non-uniform electroosmotic flow; and, (2) adsorption of the neutral dye to the walls. By comparing data to model predictions in the presence or absence of adsorption/desorption kinetics, Garcia *et al*. point out that for *h*/λ_*D *_< 4 reaction kinetics with the walls can be significant.

There is another important factor that deserves attention at this stage. It has been shown that in micron-scale channels wall adsorption/desorption kinetics may have a deleterious effect on observable dispersion [22-25]. This is the case when the net surface reaction kinetic rates are slow compared to (or on the order of) net axial transport. In nanochannels, and adsorption/desorption kinetics are intrinsically more important as the surface to volume ratio increases[26,27]. This is reflected in two facts: (1) the mass lost to the walls is more important as the channel dimension is reduced, because the number of molecules in solution is smaller; and (2) diffusive transport becomes more efficient – a decreased Damkohler number.

It is also easy to imagine that surface roughness and/or variations in the channel depth may strongly affect resolution in nanochannel electrophoresis. Although these effects have not been studied extensively there is indication that a rough surface or variations in the channel profile effectively increase the distance an ion near the wall must travel while subject to a smaller field with respect to an ion in the bulk of the flow[28,29]. We may reasonably expect a degradation of the separation efficiency for at least roughness elements of a few percent of channel depth or higher.

Next, consider the case of nanochannel electrophoresis of macromolecules. First, when the largest molecule dimension becomes on the order of the channel depth, we understandably expect steric interactions with the channel walls to influence molecule diffusion, orientation, and time-averaged locations within the channel. Evidence of this is the occurrence of electrokinetic separation of DNA fragments (100–1000 base-pair) in channels 100–300 nm deep[18]. Through steric interactions, macromolecule shape plays a strong role in its observable drift motion. Steric effects may even strongly affect adsorption/desorption kinetics. Second, a 10^7 ^V/m transverse field is strong enough to cause significant polarization of at least some macromolecules. For example, DNA of 12 kbp-10 Mbp length are known to polarize under 1000–5000 V/m field[30] and avidin (25 kDa) is known to polarize at 10^6 ^V/m fields[31]. Polarization forces therefore may add complexities as they can presumably induce anisotropic diffusivity and influence the rate of collision with channel walls. The counterion layer that surrounds a charged macromolecule can itself appreciably change the mobility, steric interactions, and diffusivity especially when the background electrolyte concentration is low. The thickness of the EDL directly influences the shape of the molecule, its stiffness, and its hydrodynamic and electrostatic screening lengths. Clearly, for macromolecular transport, continuum models fail and we will need at least Brownian dynamic simulations and perhaps atomistic simulations to predict electrophoretic transport even to first order.

Recent experimental evidence, supported by some theoretical and numerical investigations, also highlights very interesting phenomena that arise at the interface between micro and nanochannels[6,7,32,33]. These are a consequence of the dominant presence of EDL in the nanochannel. Ion-enrichment and ion-depletion zones can be generated in the vicinity of intersections of a microchannel and a nanochannel[6,33,34]. The phenomenon is not completely understood at present, but a common qualitative description that has appeared in the literature points to the increased flux of ions within the nanochannel due to the enhanced transport within the EDL. The ionic flux imbalance between the micro and nanochannels is such that regions are formed near the inlet and/or outlet of the nanochannel where the ionic strength is relatively low with respect to the bulk. Under these conditions (and after significant enrichment and/or depletion) two effects couple. First, the EDL thickness increases because of the lower ionic strength and vice versa. Second, ions are effectively shielded from entering the nanochannel, because of the extended EDL, and they accumulate (stack) at the boundary of the EDL. This behavior has been used to preconcentrate proteins and peptides to a reported level of 1-million fold[8].

Lastly, as mentioned at the beginning of this section, the dynamics of a concentrated solute can couple with that of the background electrolyte ions to affect the local EDL and EDL-associated liquid velocities in a transient manner. The description in these cases become exceedingly difficult, and so far has been intractable.

## Potential implications of nanochannel electrophoresis and opportunities in DNA separation electrophoresis

There are at least a few potential implications of the nanochannel physics discussed above. Small ions migrate through the EDL at rates determined partly by their valence. Knowledge of the bulk electroosmotic flow (e.g., measured using a neutral fluorescent species) can be exploited to accurately measure ion-valence via electrophoresis in nanochannels. This concept was discussed by Pennathur and Santiago[3,4,35,36] who also introduced a method they called electrokinetic separation by ion valence (EKSIV) which allows the independent measurement of ion-valence and ion-mobility by comparing transport properties measured in micro- and nanochannels. This implies that in nanochannels, at least relatively small molecules with a weight-dependent charge are transported through the EDL at speeds that are themselves dependent on valence/molecular weight. Such may be a mechanism for separation. This seems to be the case for DNA oligonucleotides where it has been shown experimentally that 1–100 base pair molecules can be separated, breaking the well-known parity of scales between viscous drag and electrical force[37]. Protein electrophoresis is another important potential application[38]. Proteins display a number of acidic or alkaline groups on their surface as result of amino acid sequence and the protein ternary structure. It is thought that the products of cell lysis will transport in similar fashion through the EDL, and this speculation has spurred a number of experimentations in the field of free-solution proteomics[38]. Perhaps the lysed contents of the cell nucleus can be analyzed with high-fidelity and non-destructive gel-free electrophoresis in nanochannels. This in fact part of a more ambitious vision which we might call nanochannel electrochromatography, which can perhaps implement fast and accurate separations based on the coupling of all the physics described above.

Perhaps the clearest potential application of nanochannel electrophoresis are potential improvements to the state of the art of DNA separation and sequencing. Conventional methods of DNA electrophoresis make use of either a gel or a concentrated solution of hydrophilic polymers as a separating medium in which DNA molecules migrate in the presence of an electric field [39-43]. Gel-less separation of DNA via nanochannel electrophoresis, for example, might offer a significant reduction in both cost and time across a wide range of basic research, medical, and forensics applications[38,40].

There has been work in applying nanometer scale devices for DNA separation[44]. Methods such as entropic-trapping [45-47] or ratchets [48-53] or the use of nanopores [54-58], rely exclusively on steric-type interactions for separation. We have discussed how at these scales there are relevant and important coupling with several other key physical effects, most notably "long range" electrostatic coupling within a few double layer length scales in addition to steric effects. Nanochannels are potentially a way to leverage such couplings by establishing lucrative equilibria and by optimizing the interactions to achieve specific separations. Gel-less electrokinetically-driven separation of DNA has been shown[18,37,59]. The bulk of available data is for electrophoresis of 1–100 base-pair DNA oligonucleotides in depths of 40 nm, 100 nm and 1560 nm[37]. Using these results and arguments from EKSIV-type theory, the latter paper argues that there is a complex interplay between finite-size effects (responsible for steric interactions and excluded volume effects), the ionic screening of DNA molecules, the ionic strength of the suspending electrolyte, the electrolyte/wall EDL, and the applied electric field. The physics of the situation are rich.

## Outlook

Coupling of disparate physical forces at the nano-scale allows for unique functionality in separation science. Electrophoresis in nanochannels is a clear method to exploit such coupling in free-solution fluidic devices for fast and accurate electrophoresis and chromatography.

To achieve this goal it is important at this stage to develop a fundamental understanding of how each phenomenon is regulated, and how the coupling of these affects observable separations. There is an immediate necessity to experimentally probe the dynamics of electrophoretic separations in nanochannels, to expand the as-yet-limited knowledge base. Experimental observations can potentially highlight the dominance of one effect over the rest and even systematically map the observable physics. In our minds, these efforts should be undertaken with a goal of discovering novel functionality in separation science.

**Table 1:** Simple classification of referenced work

**Fundamental studies in nanochannels**	
Experimental	[1–9, 17, 18, 32–34, 37, 39–44, 46–48, 50, 54–58]
Theory/Simulations/Review	[3, 4, 10–16, 19–31, 35, 36, 38, 49]

**DNA separations in nanochannels**	

Experimental	[1–9, 18, 32–34, 37, 39–48, 50–59]
Theory/Simulations/Review	[3, 4, 10–16, 19–31, 35, 36, 38, 49]
